# Patients’ perspectives on the implementation of intra-dialytic cycling—a phenomenographic study

**DOI:** 10.1186/1748-5908-7-68

**Published:** 2012-07-25

**Authors:** Susanne Heiwe, Helena Tollin

**Affiliations:** 1 Department of Medicine & Department of Clinical Sciences, Division of Nephrology, Karolinska Institutet, Stockholm, Sweden; 2 Department of Renal Medicine, Haemodialysis Unit, Karolinska University Hospital, Stockholm, Sweden

**Keywords:** Chronic kidney disease, Exercise, Health behaviour, Renal replacement therapy, Behavioural change

## Abstract

**Background:**

Adults undergoing haemodialysis have significantly reduced physical capacity and run a high risk of developing cardiovascular complications. Research has shown that intra-dialytic cycling has many evidence-based health effects, but implementation is rare within renal clinical practice. This may be due to several causes, and this study focuses on the patients’ perspective. This perspective has seldom been taken into account when aiming to assess and improve the implementation of clinical research. The aim of this study was to describe how adults undergoing in-centre haemodialysis treatment experienced an implementation process of intra-dialytic cycling. It aimed to identify potential motivators and barriers to the implementation process from a patient perspective.

**Methods:**

Maximum-variation purposive sampling was used. Data were collected until saturation, through semistructured interviews, which were analysed using phenomenography.

**Results:**

The implementation of intra-dialytic cycling was experienced as positive, as it had beneficial effects on physical and psychological well-being. It was easy to perform and did not intrude on patients’ spare time. These factors increased the acceptance of the implementation and supported the maintenance of intra-dialytic cycling as an evidence-based routine within their haemodialysis care. The patients did, however, experience some barriers to accepting the implementation of intra-dialytic cycling. These barriers were sometimes so strong that they outweighed the participants’ knowledge of the advantages of intra-dialytic cycling and the research evidence of its benefits. The barriers sometimes also outweighed the participants’ own wish to cycle. The barriers that we identified concerned not only the patients but also the work situation of the haemodialysis nurses.

**Conclusions:**

Consideration of the motivators and barriers that we have identified can be used in direct care to improve the implementation of intra-dialytic cycling.

## Background

Chronic kidney disease is a worldwide public health problem: the average life expectancy of patients undergoing haemodialysis is a quarter of that of healthy age-matched individuals [1]. Reduced physical capacity is one of the main stressors of these patients [2-4]. The physical capacity of adults undergoing haemodialysis treatment is reduced to such an extent that it impinges on their ability and capacity to perform activities in everyday life [3-6]. The National Kidney Foundation Disease Outcomes Quality Initiative (NKF K/DOQI) guidelines [7] stress that physical exercise should be seen as one of the cornerstones of renal therapy. It is, therefore, important that interventions whose benefits have been demonstrated are implemented within renal clinical practice.

Regular physical exercise by this group of patients significantly reduces cardiovascular risk factors. It improves physical capacity and psychological well-being [5,8]. Exercise training is, however, seldom implemented in renal self-care. One reason is that patients find that they do not have time. They have a strict haemodialysis schedule of three to five treatment sessions per week (each of duration four to five hours), and extreme fatigue often follows a session, causing the patients to sleep for the rest of the day [3,4,9]. This forces them to set priorities among their self-care activities, and they deselect self-care activities whose effects are beneficial, such as regular exercise training [4,10].

This lack of time is a well-known barrier to exercise, and thus researchers have started to study the effects of intra-dialytic cycling. Present evidence indicates that intra-dialytic exercise can mitigate many of the primary independent factors for early mortality in end-stage renal disease [1]. Intra-dialytic exercise is a beneficial exercise intervention that improves physical fitness, haemodialysis efficiency, and nutritional well-being. It also reduces fatigue and has beneficial effects on inflammation status, psychological status, and health-related quality of life [6,11-33]. Intra-dialytic cycling within clinical practice should be easy to implement within renal care as it takes place during the haemodialysis treatment and should not, therefore, cause a feeling of lack of time.

Despite the documented benefits of intra-dialytic exercise training for adults undergoing haemodialysis treatment, most haemodialysis clinics have not introduced exercise for their patients. There may be many reasons for this, including a lack of patient interest and a lack of knowledge about its beneficial effects [34]. The Promoting Action on Research Implementation in Health Services (PARiHS) program has developed a conceptual framework that tries to describe the complexities around the art of implementation. This is divided into three main elements: evidence, context, and facilitation [35]. We chose to focus on potential barriers and motivators for the implementation of intra-dialytic cycling in clinical practice from a patient perspective. Patient involvement is fundamental to achieving the successful implementation of evidence-based therapy [36]. Internal personal perceptions may inhibit a person’s involvement in physical activity, as these perceptions may cause the individual to be fearful of engaging in such activity or cause him or her to lose the interest or the desire to engage in it [37]. On the other hand, internal personal perceptions of positive feelings of well-being and self-efficacy may promote a person’s involvement in physical activity [37]. It is important to increase our knowledge of how patients experience the implementation of intra-dialytic cycling, in order to identify motivators and barriers. This knowledge can subsequently be used to create clinical strategies that encourage and support the initiation and maintenance of regular intra-dialytic exercise among adults undergoing haemodialysis treatment. This article is a contribution to this increase in information.

## Aim

The aim of this study was to describe how adults undergoing in-centre haemodialysis treatment experienced the implementation of evidence-based intra-dialytic cycling, in order to identify potential motivators and barriers to continued intra-dialytic cycling as part of patients’ routine nephrological care.

## Methods

### Design and setting

The study asked patients to describe their experiences of the implementation of intra-dialytic cycling at a haemodialysis unit (in Stockholm, Sweden). A qualitative research method was used to identify patient preferences. This approach enhances knowledge of health and healthcare [38] and is useful to achieve a more in-depth understanding of a phenomenon [39]. We have used a phenomenographic approach in the study presented here. Phenomenography seeks to define, describe, and analyse people’s experiences and conceptions regarding a phenomenon [40,41]. The key results of phenomenography are its outcome space and its descriptive categories [41], which represent the different ways in which the phenomenon is understood [42].

### Implementation of the intervention

All physicians and nurses at the haemodialysis unit received information and education concerning intra-dialytic cycling and its beneficial effects. They were informed that the intervention should be seen as a part of the routine care at the haemodialysis unit. They were asked to encourage the patients during the intra-dialytic cycling and to try to make them feel that they were receiving positive attention from the staff. The physiotherapist who provided information, education, and support to the staff (and thus worked as a ‘facilitator’) also provided individual information, education, and support to each patient. The physiotherapist created a cycle that could be easily placed at the end of the patient’s bed or dialysis chair. The cycle was tested in a pilot experiment on four patients and modified based on their comments. The intra-dialytic cycling consisted of 30 minutes of intra-dialytic cycling at an intensity of 13–15 on the ratio of perceived exertion (RPE) scale. The physiotherapist provided oral encouragement, as did the haemodialysis nurse and, when present, the renal physician. Data were subsequently collected through patient interviews dealing with the patients’ experiences of the implementation of intra-dialytic cycling within their clinical haemodialysis treatment program.

### Sample

Data were collected from a purposive sample of adults who were exposed to intra-dialytic cycling at a particular in-centre haemodialysis unit in Sweden. Inclusion criteria were that participants had to be adults undergoing regular haemodialysis treatment. Exclusion criteria were as follows: non-Swedish speaking or physical or functional inability to perform 30 minutes intra-dialytic cycling (this ability was assessed by the renal physiotherapist). Maximum-variation purposive sampling was used. Patients were classified according to the following strata:

· men–women

· duration of haemodialysis treatment <9 months—5 years— >10 years

· young (18–30 years)—lower middle-age (31–45 years)—upper middle-age (46–65 years)—elderly (66–80 years)—old (81 years and older)

· sedentary—irregular physical activity—regular physical activity.

The physiotherapist assessed the patients’ ability to participate in intra-dialytic cycling. The author (HT) then contacted suitable participants using the list of strata and the inclusion and exclusion criteria. The study protocol was approved by the local Ethics Committee. Patients were informed orally and in writing, and informed consent was obtained from all participants at each interview session.

No patients at the haemodialysis unit were below 46 years of age, and none needed help with mobility at the time of recruitment. The demographic data were as follows: six men and four women; duration of haemodialysis treatment 11 months–157 months; age 54–81 years; sedentary (n = 3), irregular physical activity (n = 3), and regular physical activity (n = 4); haemodialysis treatment in bed (n = 5), haemodialysis treatment in chair (n = 5); haemodialysis treatment in a room for two patients (n = 3) or in a room for eight patients (n = 7). The youngest patient was 54 years of age, while the mean age was 66.5 years.. All participants came from the Nordic countries. Saturation was reached after 10 interviews, meaning that no new information was collected, and no more patients were interviewed.

### Data collection

Data were obtained by audiotaped, semistructured interviews of duration 30–90 minutes, which were transcribed verbatim. The participants chose when and where the interviews took place. Eight participants decided to be interviewed during a haemodialysis session and two on other occasions. The interviews were conducted by HT at the haemodialysis unit. Patients were encouraged to develop their thoughts about the phenomenon in question as freely as possible. The first question was, “How did you experience cycling in bed/the haemodialysis treatment chair during your haemodialysis treatment?” Subsequent questions were adjusted to each patient’s response. Two pilot interviews were performed and the interview technique and the interview guide adjusted slightly.

### Data analysis

Data were analyzed according to a contextual analysis within a phenomenographic approach [43]. A contextual analysis provides both categorizations and relationships between these categorizations in the form of combinations or patterns of categories.

1. The transcribed interviews were read repeatedly without structuring to enable the researchers to become familiar with the content and gain a sense of the whole.

2. Statements irrelevant to the phenomenon were discarded from the interviews. Differences and similarities in the participants’ experiences were noted by contrasting and comparing excerpts from all interviews with one another. A preliminary pattern of descriptive categories was constructed. The categories represented different experiences of intra-dialytic cycling.

3. The preliminary pattern of categories was scrutinised and subsequently revised to give a final pattern of categories. These categories differed distinctively and qualitatively from each other. The final descriptive categories were placed into the outcome space.

4. The internal relationships between the categories were described in order to study how the different categories interacted with one another.

### Rigour

Malterud’s research quality guidelines for qualitative research were used [38]. Reflexivity was obtained by examining data, or their interpretations, for competing conclusions. The participants were recruited strategically using a list of strata in order to ensure transferability. The systematic process of collecting data and analysing the material was thoroughly described to ensure that the process can be shared with others. Four probing strategies were used in order to make sure that the study result gave the participants’ conceptions rather than the researcher’s. These strategies were repeating, requesting clarification, requesting elaboration, and requesting confirmation. It is necessary in phenomenographic research that another researcher (in this case, SH) recognises the descriptive categories once they have been identified [41]. We agreed readily on the results of the data analysis in the present study.

## Results

The findings identify factors that can function as motivators or barriers to patients making a decision to accept and support the implementation of intra-dialytic exercise or not. The implementation strategy was successful in that all patients wanted to continue with intra-dialytic cycling as a part of their routine haemodialysis care. Intra-dialytic cycling was experienced as positive due to its beneficial effects on patients’ physical and psychological well-being, due to it being easy to perform, and due to the fact that it did not intrude on patients’ spare time. These factors increased acceptance of the implementation and supported the maintenance of intra-dialytic cycling as an evidence-based routine within patients’ haemodialysis care. The patients did, however, also describe various barriers to the implementation of intra-dialytic cycling. These barriers were sometimes so strong that they in some cases outweighed the participants’ theoretical knowledge of, and research evidence for, the advantages of intra-dialytic cycling and sometimes also a participant’s wish to cycle. The barriers that were identified related not only to the patient himself or herself but included also concern for the work situation of the haemodialysis nurses.

**Figure 1:**
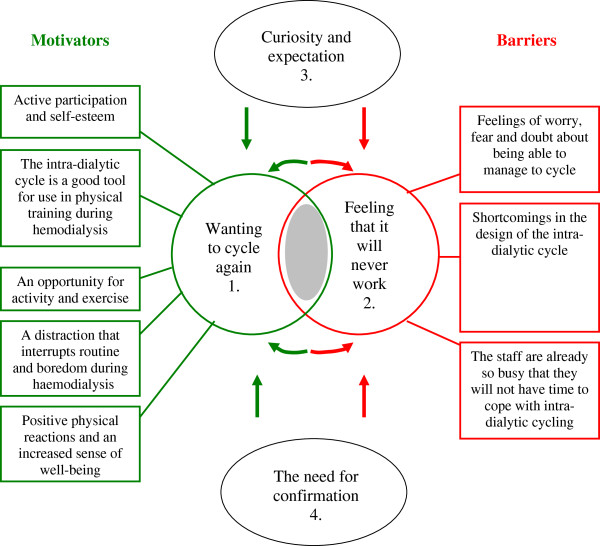
**The descriptive categories and their inter-relationship.** Green (circle 1 and its rectangles) symbolises motivators, while red (circle 2 and its rectangles) symbolises barriers. The grey zone between circles 1 and 2 represents the individuals who are considering accepting the implementation of intra-dialytic cycling (motivators) or not (barriers). The descriptive categories 3 and 4 are factors that can either work as motivators (and therefore bring the patients closer to circle 1 or cause them to remain in circle 1) or have the opposite effect and reinforce barriers (and therefore take the patient out from circle 1 towards circle 2 or make the patient more convinced to remain in circle 2). The particular effects of the factors in ovals 3 and 4 depend on the surrounding environment.

The descriptive categories that we identified are presented below, and the relationships between them are discussed. Each descriptive category is exemplified by a quotation. Figure 1 presents the outcome space, in which all descriptive categories are located.

### The design and function of the intra-dialytic cycle

The needles in the arteriovenous fistula/graft must not be moved while exercising. It was hypothesised that this would be a factor that would scare the patients and discourage them from performing intra-dialytic cycling, even though the implementation of the intervention included information that there were no risks for their arteriovenous fistula/graft. However, none of the participants related that they had experienced any problems with this. They described how the design and function of the cycle were more important to them. Most patients experienced the cycle as easy to handle without assistance and, thus, regarded it as a useful tool for exercising while undergoing haemodialysis. “I was surprised how easy it was to cycle right there in bed.” The phase of the implementation during which the cycle was designed and modified based on pilot patients’ opinions about the cycle facilitated successful implementation.

Some patients experienced *s*hortcomings in the design of the intra-dialytic cycle. Those who cycled energetically complained that the cycle was not completely still while cycling. Some found it difficult to keep their feet on the pedals, while others found the pedals to be inflexible. These experienced shortcomings functioned as barriers to intra-dialytic cycling and had a negative impact on the implementation process.

"*“Well, it wasn’t quite firm, it moved around a bit….”*"

### Active participation and self-esteem

The participants felt a high internal locus of control for three reasons. Firstly, they felt that they were taking an active part in their care, and secondly, they had an opportunity to decide when to exercise during haemodialysis. Finally, they could control the resistance of the cycle. The participants were not informed before the intervention that intra-dialytic cycling is much easier than is submaximal ergometer-cycle tests. Those who had undergone submaximal ergometer-cycle tests were, therefore, sceptical of the implementation. These patients described how they had undergone submaximal ergometer-cycle tests and experienced them as extremely physically demanding and, hence, were against all forms of exercise. All patients, however, were obliged to try intra-dialytic cycling at least once as a part of the implementation and to describe their experience. The sceptical patients now experienced relief, as the intra-dialytic cycling was experienced as being much easier. It was evident that experiences from earlier submaximal ergometer-cycle tests were a barrier to accepting the implementation of regular, evidence-based intra-dialytic cycling and that information about this should be included in the implementation of the intervention, together with pilot cycling for all patients. These measures will increase the probability of successful implementation.

"*“I thought it was good with the resistance as you could regulate it as you saw fit.”*"

### An opportunity for physical activity and exercise

Patients regarded the implementation of intra-dialytic cycling as positive. They were all aware of the importance of regular exercise for those with chronic kidney disease and became even more so after having been informed and educated by the physiotherapist. They were positive about the idea of exercise but described how it was impossible to add exercise into their daily life. They wanted to increase the amount of exercise they undertook, in order to increase muscular strength, but suffered from time constraints. The implementation of intra-dialytic cycling was, therefore, appreciated, as it gave them the opportunity to use their time in haemodialysis to exercise. They regarded intra-dialytic cycling as a way of saving valuable nondialysis time, which could be used for other pleasurable activities and rest. Intra-dialytic cycling gave them more free time.

"*“I wouldn’t need to come into the hospital on my free days [to exercise]…I think it’s a good way to get regular exercise as it’s not so easy to do it alone at home.”*"

### A distraction that interrupts the routine and boredom during haemodialysis

Most patients undergoing haemodialysis treatment find the time on dialysis to be boring and monotonous. It was suggested to patients in the implementation of the intervention that the cycling could be seen as something active and pleasant while on haemodialysis. This motivated the patients to try intra-dialytic cycling. The patients reported the intra-dialytic cycling to be a welcome distraction, something that interrupted the routine and made time pass more rapidly. This experience increased patients’ acceptance of the implementation and functioned as a facilitator, as the patients talked about this experience with other patients, including those who were sceptical to the implementation.

"*“It’s easier to bear the time you’re chained to the machine if you’ve got something to look forward to, something to pass the time …cycling for example.”*"

### Curiosity and expectation

Most participants described how they became curious about the implementation of intra-dialytic cycling at an early stage, when the physiotherapist informed them about it. Most participants looked forward to trying it. Providing information about the beneficial effects of evidence-based intra-dialytic cycling had a beneficial effect on the implementation process."Patients described how they had heard about the project through contact with patient organisations, and this facilitated the implementation process. Patients were curious to see how well they would perform. Patients expected that cycling would increase muscular strength.

"*“Yes, I was a bit curious. Curious and expectant perhaps, about what it would lead to.”*"

### The need for confirmation

The participants expressed the need to have someone to talk to while cycling. The implementation of the intervention included the physiotherapist being present while a patient cycled, and this increased patients’ acceptance of intra-dialytic cycling. The need to be seen as an individual and receive oral confirmation and the need to have behaviour reinforced and encouraged by the staff were both important. Reinforcement by staff could be indirect (by positive body language, for example) or direct (“Yes, go for it, you can make it!”). The belief that the physiotherapist would not be able to continue to attend sessions in order to assist and encourage the cycling was a barrier that had a negative impact on the success of the implementation in the long run.

"*“The staff came in and were a bit curious.... You felt like the centre of attention for a while.”*"

Despite the importance of being confirmed as an individual, few participants reported that they had received comments, questions, or reactions from any of the other patients in the room. Some acknowledged that they had not asked other patients about their experiences but pointed out that the placement of the beds and haemodialysis chairs in the room was not optimal for conversation. This had a negative impact on acceptance and support for the implementation in the long run, and this needs to be considered in the implementation of the intervention.

### Doubtful thoughts and emotions

Patients had been informed by the physiotherapist, as part of the implementation of the intervention, that the workload would be sufficiently small for all patients to be able to cycle for 30 minutes. Patients, however, reported feelings of worry, fear, and doubt that they would not have the physical capacity required to cycle for 30 minutes and would have to interrupt the session. Some patients had problems with lower or upper back pain and were concerned that cycling in bed or the haemodialysis chair would increase their pain. The participants also worried that they would experience cycling as boring in the long term, and this would cause them to drop out. Some thought of cycling as a strenuous activity and feared muscle tiredness. These factors all worked against acceptance and continuation of the implementation and should be taken into account in a modified implementation of the intervention.

"*“You wondered if they were going to make it really tough....”*"

### Physical reactions and a sense of well-being

All patients were obliged to try intra-dialytic cycling at least once, and this facilitated the implementation. The long period of immobilization during haemodialysis treatment often causes stiffness. Patients reported that this problem was reduced after their first session of intra-dialytic cycling. They also expressed satisfaction that their physical well-being increased. The reduced stiffness, retained mobility, and increased well-being functioned as facilitating experiences. Patients did not feel that the exercise period of 30 minutes was unduly long (despite their initial anxiety of not being able to manage as long as 30 minutes). This gave rise to feelings of satisfaction and pride in having cycled for so long. These positive experiences created a situation in which patient preferences facilitated and supported the maintenance of the implementation as routine clinical practice.

Some patients experienced various physical symptoms during and after cycling. Patients found it easier to lie down while cycling than to sit on a conventional ergometer cycle, which in some cases was associated with pain and problems with balance. Furthermore, problems with breathlessness while exercising were less problematic while cycling in bed or a haemodialysis treatment chair. Leg cramps occurred while cycling but were not related to the cycling itself, being described by patients as a usual symptom during dialysis. Indeed, some patients experienced fewer leg cramps and feelings of restless legs after having cycled. Most felt acute muscular discomfort (lactic acid) in the legs after 10 minutes of cycling. They were glad that the physiotherapist was standing next to them and encouraged them to continue cycling, as the acute muscular discomfort passed. Patients were surprised that they could continue cycling and that this reduced the unpleasant discomfort (lactic acid), and these experiences facilitated the implementation process. Muscular fatigue was experienced immediately after the cycling session but disappeared after 15 to 30 minutes. The muscles were stiff on the day after the intra-dialytic cycling, but patients did not feel restricted by this. No negative physical symptoms were experienced during or after the cycling, and thus patients did not experience their physical symptoms as a barrier to accepting the implementation. This descriptive category emphasises the importance of having all patients attempt the cycling at least once, as part of the implementation of the intervention.

"*“You didn’t have to sit and tense yourself… just sit calm and relaxed and use your legs…I didn’t feel anything when I sat like this in bed and cycled… that was the good thing about it, I felt no strain at all on my back.”*"

### Concern for the work situation of the healthcare providers

The implementation of the intervention did not include providing any information to the patients about how the implementation would affect the work situation of the healthcare providers. The participants, however, expressed concern that the work situation of the healthcare providers would be negatively affected by the implementation of intra-dialytic cycling. They did not want to burden the staff and expressed worry that evidence-based intra-dialytic cycling would cause stress for the staff, as it would probably be time consuming and energy consuming to place the cycle at the end of the patient’s bed. Patients did not want to burden the staff, and this attitude was thus a barrier to the implementation process. The patients considered the cycle to be heavy, and they perceived this as an obstacle for staff. The idea that cycling was a burden for nurses became another barrier for successful implementation, even though the participants never heard the staff mention this, and the cycle had been designed to be easy for the staff to handle (with the aid of wheels and a lifting system). Evidently, it is important to modify the implementation of the intervention so that it provides information to the patients that can remove these ideas at an early stage of the process.

"*“…you [the staff] get a sort of extra job when you have to set up the bike and adjust it to each patient…”*"

### Internal relationships between the descriptive categories

The results revealed internal relationships between the descriptive categories (see Figure 1). The implementation strategy, for example, reinforced the patients’ curiosity and expectations and their need for confirmation, such that the staff could increase the patients’ acceptance of the implementation and increase support for the subsequent maintenance of the implementation within clinical practice. The results, however, also revealed the opposite effect, and the actual effect achieved depended on the staff’s interactions with the patients. If the staff ignored patients’ needs for confirmation and/or curiosity and expectations, patients who were not already motivated and physically active experienced difficulty in accepting the implementation. Further, those who were already motivated and physically active risked a relapse in the process of their behavioural change and, in the long term, the risk arose that they would not continue to accept and support the implementation. This information must be used in a modified version of the implementation of the intervention for intra-dialytic cycling.

## Discussion

Even though physical exercise is a beneficial activity, and despite physical activity being included in the guidelines for renal care [7], exercise is rarely implemented within renal care. The factors that determine whether research results are taken up in healthcare practice are poorly understood, but awareness is growing that organisational factors are important [44]. The PARiHS framework suggests that there are three key elements to successful implementation: evidence, context, and facilitation [35,45]. Cumming’s findings highlight the combined importance of culture, leadership, and evaluation in increasing the clinical use of research results [44], thereby strengthening the PARiHS framework. Bayliss *et al.* (2006) showed that intra-dialytic exercise can become a reality and a standard treatment for patients undergoing haemodialysis, provided that the administration and staff are fully committed [34]. The present study has also identified commitment as an important factor for successful implementation. The implementation of the intervention presented here included a continuous educational programme designed to create an organisational readiness for change among both the staff and the patients. Organisational readiness can be described as a shared psychological state in which members feel committed to implementing change and feel confident that they can accomplish the implementation [46]. Weiner (2009) has shown that the degree of organisational readiness for change depends on how much patients and staff value the change and how favorably they appraise task demands, resource availability, and situational factors. The higher the degree of organisational readiness among patients and staff, the more effective will be the implementation [46]. The implementation of the intervention in the present study should be modified to increase the degree of organisational readiness among the patients. One example is that the patients must develop greater confidence that they can accomplish 30 minutes of intra-dialytic cycling, without the fear of becoming exhausted. Gagliardi *et al.* (2011) have also discussed the importance of clinical guidelines and present many ways in which these can be modified to facilitate their use [47]. It is also important to identify and understand factors that function as barriers to and motivators for the implementation process, from a patient’s perspective. Diaz Del Campo *et al.* (2011) stress that patient involvement is fundamental to achieving patient-oriented clinical practice guidelines [36]. The results of the present study highlight this importance when aiming to achieve the successful implementation of interventions and evidence-based clinical care.

The factors that influence the levels of physical activity in adults with diseases are affected not only by health status but also, and more strongly, by perceptual factors, such as perceived health status (negative and positive well-being), symptom distress, and self-efficacy [48]. Negative levels of well-being (arising from, for example, emotional distress or depression) are also an important factor associated with the levels of physical activity achieved [48]. This was also evident in the present study, as all motivators and barriers were perceptual factors.

It is important to consider the patient’s situation and his or her view of motivators for and barriers to the implementation of evidence-based exercise. This is important also when helping a patient to change his or her level of physical activity. Haemodialysis patients have a very special life situation in which haemodialysis care (including healthcare transportation, haemodialysis, physiotherapy, nutrition, and self-care activities) occupies most of their time and is perceived to impinge on patients’ limited and valuable nondialysis time [49]. This feeling was expressed by the patients in the present study. Konstantinidou *et al.* (2002) described how difficult it is to convince such patients to participate in exercise during nondialysis time, even though there is substantial evidence for the benefits of regular exercise [25]. This was evident in the present study, and the opportunity to exercise while undergoing haemodialysis saved valuable nondialysis time and, in this way, removed one of the main barriers to exercise.

Jablonski (2007) has shown that muscular weakness is one of the highest-ranked stressors among adults undergoing haemodialysis [50]. It was clear in the present study that the patients knew that their loss of muscle function was related to their chronic kidney disease and that exercise was the only treatment for it. They did not, however, find the time to exercise and felt a need for coaching due to their fatigue and fear of physical activity. Many were seriously worried about developing more negative symptoms from exercise. The implementation process, however, had a positive impact on such doubtful thoughts and emotions, since patients experienced the intra-dialytic cycling as surprisingly easy and the duration of the exercise as shorter than expected. Many elderly patients undergoing haemodialysis have problems also with their balance, and many experience lower back pain. These patients expressed a fear that the intra-dialytic cycling would increase their back pain, for example, and this fact worked against acceptance and support of the implementation process. However, most patients lost this fear after the initial intra-dialytic cycling, because they experienced it as easy to do and because their lower back pain did not increase while cycling in bed or sitting in a haemodialysis chair. Informing sceptical patients about the advantages of cycling in bed or in the haemodialysis treatment chair will enable healthcare providers to motivate such patients.

Activation is an important motivator for wanting to continue with the activity [51], while self-efficacy is a key variable that explains physical activity levels in adults [52]. Social cognitive theory [53,54] shows that there are many disincentives to being physically active and that individuals need positive rewards and high self-efficacy to overcome difficulties or barriers in order to engage in physical activity [53,54]. Self-efficacy is one of the most important constructs for assessing intermediate outcome and predicting future success [55,56]. Behavioural change is often described as passing through stages, and self-efficacy significantly and independently contributes to the passage between stages. Self-efficacy increases from the precontemplation to the maintenance stages [57,58]. This increase in self-efficacy does not depend on the scale used to measure it, nor on the population studied, showing that exercise self-efficacy is universal [59]. The results of the present study also showed the importance of self-efficacy. Intra-dialytic cycling was associated with positive well-being, feelings of being an active part of the treatment process, feelings of control and increased self-esteem, and an increase in the feeling of self-efficacy following patients’ successful attempts at intra-dialytic cycling. These factors are important in achieving successful implementation.

Patients were concerned about causing extra work for the staff, even though they had not heard any mention of this from the staff. This raises the question of what signals the staff gives. Further research of this issue is necessary, as the staff’s signals unconsciously affect patients’ attitudes to intra-dialytic cycling. The staff needs to consider how silent communication can be interpreted by patients. Silence can be interpreted positively within the Swedish culture, whereas discussion arises when something is not good. This may be misinterpreted by patients as a failure by the nurses to see them, or as expressing the opinion of the nurses that the activity is of minor importance. This conclusion is supported by previous research [51]. It is important to study the attitudes of the healthcare providers to an intervention, if it is to be successfully implemented, not just the implementation process itself. Furthermore, it is important to know an individual’s current stage in the behavioural change process, as different supporting interventions are needed at each stage [51,60]. Providing adequate advice and motivation at the correct stage of change is crucial for successful behavioural change [51,60], while inadequate advice and motivation may result in relapse [51,60].

This study has both strengths and limitations. Data were collected until saturation, but it is possible that more experiences may have been found from more interviews. This would not, however, invalidate the aspects that the present study has revealed. It is important that the study be replicated in various cultures to investigate whether further aspects can be identified.

The findings from the present study offer healthcare providers in-depth knowledge that can provide them with tools to increase patients’ acceptance and support for the implementation process. This can be achieved by reinforcing motivators and factors such as ‘curiosity and expectations’ and the ‘need for confirmation’. Furthermore, healthcare providers can reduce the impact of barriers by being aware of them, identifying them, and trying to eliminate them. Haemodialysis is experienced as boring, and intra-dialytic cycling functions as a distraction, so healthcare providers can use this to increase patient motivation for the implementation. Asking patients *when* and not *if* they want to cycle during haemodialysis reinforces patient motivation, as the cycling is then presented as a natural part of the regular renal treatment program.

## Conclusions

Patients’ reactions to the implementation of intra-dialytic cycling were mainly positive, as it made it possible for patients to save valuable nondialysis time, while using haemodialysis time for something of benefit to their health. Cycling also functioned as a distraction during haemodialysis. Healthcare providers must become aware of motivators for and barriers to patients’ acceptance and support of the implementation process in order to obtain tools that can be used to provide evidence-based care that maintains or improves patients’ physical and psychological health. Future research should examine an implementation of the intervention that has been modified based on the results presented here and assess its capacity to increase the clinical use of research results. Also, the experiences of healthcare providers of the implementation process of evidence-based intra-dialytic exercise in clinical practice must be evaluated in order to identify barriers and motivators from a staff perspective.

## Competing interests

The authors declare that they have no competing interests.

## Authors’ contributions

SH made substantial contributions to the conception and design of the study, acquisition of data, analysis and interpretation of data, drafting and revising of the manuscript, and gave final approval of the manuscript. HT contributed to the acquisition, analysis, and interpretation of data. Both authors read and approved the final manuscript.
